# MAM-E17 rat model impairments on a novel continuous performance task: effects of potential cognitive enhancing drugs

**DOI:** 10.1007/s00213-017-4679-5

**Published:** 2017-07-26

**Authors:** Adam C. Mar, Simon R. O. Nilsson, Begoña Gamallo-Lana, Ming Lei, Theda Dourado, Johan Alsiö, Lisa M. Saksida, Timothy J. Bussey, Trevor W. Robbins

**Affiliations:** 1 Neuroscience Institute, New York University Medical Center, New York, NY 10016 USA; 2 Department of Neuroscience and Physiology, New York University Medical Center, New York, NY USA; 3 Department of Psychology, University of Cambridge, Cambridge, UK; 4 MRC and Wellcome Trust Behavioural and Clinical Neuroscience Institute, University of Cambridge, Cambridge, UK; 5 Department of Health Industry Management, Beijing International Studies University, 1 Dingfuzhuang Nanli, Beijing, China; 6 Department of Neuroscience, Unit of Functional Neurobiology, University of Uppsala, Uppsala, Sweden; 7 Molecular Medicine Research Group, Robarts Research Institute, Western University, London, ON Canada; 8 Department of Physiology and Pharmacology, Schulich School of Medicine and Dentistry, Western University, London, ON Canada; 9 The Brain and Mind Institute, Western University, London, ON Canada

**Keywords:** Attention, MAM-E17, Executive function, Touch screen, Behavioral pharmacology, Schizophrenia, ADHD, Animal model

## Abstract

**Rationale:**

Impairments in attention and inhibitory control are endophenotypic markers of neuropsychiatric disorders such as schizophrenia and represent key targets for therapeutic management. Robust preclinical models and assays sensitive to clinically relevant treatments are crucial for improving cognitive enhancement strategies.

**Objectives:**

We assessed a rodent model with neural and behavioral features relevant to schizophrenia (gestational day 17 methylazoxymethanol acetate treatment (MAM-E17)) on a novel test of attention and executive function, and examined the impact of putative nootropic drugs.

**Methods:**

MAM-E17 and sham control rats were trained on a novel touchscreen-based rodent continuous performance test (rCPT) designed to closely mimic the human CPT paradigm. Performance following acute, systemic treatment with an array of pharmacological compounds was investigated.

**Results:**

Two cohorts of MAM-E17 rats were impaired on rCPT performance including deficits in sensitivity (*d*′) and increased false alarm rates (FARs). Sulpiride (0–30 mg/kg) dose-dependently reduced elevated FAR in MAM-E17 rats whereas low-dose modafinil (8 mg/kg) only improved *d*′ in sham controls. ABT-594 (5.9–19.4 μg/kg) and modafinil (64 mg/kg) showed expected stimulant-like effects, while LSN2463359 (5 mg/kg), RO493858 (10 mg/kg), atomoxetine (0.3–1 mg/kg), and sulpiride (30 mg/kg) showed expected suppressant effects on performance across all animals. Donepezil (0.1–1 mg/kg) showed near-significant enhancements in *d*′, and EVP-6124 (0.3–3 mg/kg) exerted no effects in the rCPT paradigm.

**Conclusion:**

The MAM-E17 model exhibits robust and replicable impairments in rCPT performance that resemble attention and inhibitory control deficits seen in schizophrenia. Pharmacological profiles were highly consistent with known drug effects on cognition in preclinical and clinical studies. The rCPT is a sensitive and reliable tool with high translational potential for understanding the etiology and treatment of disorders affecting attention and executive dysfunction.

**Electronic supplementary material:**

The online version of this article (doi:10.1007/s00213-017-4679-5) contains supplementary material, which is available to authorized users.

## Introduction

Neurological and psychiatric disorders such as schizophrenia, attention deficit hyperactivity disorder and Alzheimer’s disease have profiles of cognitive impairment that are critical targets for therapeutic remediation. Central to these profiles are deficits in executive function including impairments in sustained goal-directed action, attentional and inhibitory control and regulation of processing speed (Buchanan et al. 1997; Bilder et al. 2000; Knowles et al. 2010). The establishment of robust preclinical models that appropriately translate prominent cognitive features of a disorder, along with the development of valid behavioral assays that are sensitive to model impairments as well as clinically relevant treatments, is an important goal for improving cognitive enhancement strategies.

A widely used model of neurodevelopmental hallmarks of schizophrenia involves administration of the mitotic neurotoxin methylazoxymethanol acetate (MAM) to pregnant rat dams at gestational day 17. The offspring of MAM-treated dams (gestational day 17 methylazoxymethanol acetate treatment, MAM-E17) show pronounced neurodevelopmental alterations within limbic and cortical brain structures (Moore et al. 2006). Histological and electrophysiological evidence reveals structural and functional disruptions predominantly within hippocampal and frontostriatal circuitries in the MAM-E17 model (Gourevitch et al. 2004; Penschuck et al. 2006; Moore et al. 2006; Lodge and Grace 2008; Matricon et al. 2010; Hradetzky et al. 2012; Gastambide et al. 2012; Phillips et al. 2012; Snyder et al. 2012). These disruptions mirror well-established abnormalities in individuals afflicted with schizophrenia: hippocampal and cortical disorganization including aberrant expression of markers for inhibitory (Benes et al. 1996; Lewis 2000) and excitatory (Deakin et al. 1989; Tsai et al. 1995) signaling efficacy and striatal dopamine (DA) hyperfunction (Abi-Dargham et al. 2000).

The MAM-E17 model also displays several behavioral characteristics reminiscent of cognitive impairments observed in schizophrenia, including deficits in classic paradigms such as prepulse inhibition and latent inhibition (Flagstad et al. 2005; Moore et al. 2006). MAM-E17 rats show impaired prefrontal cortical-dependent reversal and extra-dimensional shift learning on a bowl-digging task (Featherstone et al. 2007; Gastambide et al. 2012) and deficits in hippocampal-dependent spatial memory on maze navigation tasks (Gourevitch et al. 2004; Featherstone et al. 2009; Gastambide et al. 2015). Impairments in inhibitory control have also been observed as decreased efficiency on a differential reinforcement of low-rate (DRL-20) task and nonsignificant trends toward increased premature responding in the five-choice serial reaction time task (5-CSRTT) (Featherstone et al. 2007). Some patients diagnosed with schizophrenia show potentially analogous cognitive impairments when challenged with computerized neuropsychological tests (Hutton et al. 1998; Birkett et al. 2007; Leeson et al. 2009; Nuechterlein et al. 2015).

One cognitive paradigm that is particularly sensitive for detecting reliable impairments in schizophrenia patients is the continuous performance test (CPT) (Wohlberg and Kornetsky 1973; Buchanan et al. 1997). Typical versions of the CPT require subjects to visually attend to a single location on a monitor and report briefly presented target stimuli amongst a stream of nontarget stimuli. Numerous cognitive/executive processes are required for successful CPT performance including attention (alerting, selective, or sustained), memory (targets versus nontargets), inhibitory control (withholding inappropriate responses), and processing speed (rapidly identifying targets). Schizophrenia patients display impairments in the CPT paradigm that appear independent of symptom severity (Nuechterlein et al. 2015) as well as clinical (Wohlberg and Kornetsky 1973; Cornblatt et al. 1997) and medication (Cornblatt et al. 1997; Keefe et al. 2006; Nuechterlein et al. 2015) status. Despite consistent CPT impairments observed in schizophrenia and robust frontostriatal alterations in MAM-E17 animals, behavioral effects in the MAM-E17 model using rodent sustained attention paradigms have been equivocal. Limited evidence of disrupted performance in MAM-E17 rats has been detected in either the 5-CSRTT (Featherstone et al. 2007) or the sustained attention task (without or including distractors (SAT/dSAT)) (Howe et al. 2015), even following extensive parameter manipulations taxing attentional and impulsive processes.

The current study implemented a novel touchscreen-based rodent continuous performance task (rCPT) (Kim et al. 2015). The rCPT closely emulates the task structure and main cognitive requirements of common variants of the human CPT by incorporating a continuous succession of several luminance-matched target and nontarget images that require both detection and discrimination as well as response inhibition on nontarget trials. We hypothesized that the rCPT might thus be more sensitive for detecting impairments in attentional and/or executive function in the MAM-E17 model. We further evaluated the impact of eight acute systemic treatments of compounds that affect a variety of neurotransmitter systems and have demonstrated potential to enhance cognitive performance in rodents and/or have efficacy for remediating impairments in clinical patient groups.

## Methods

### Animals

Male offspring of Sprague Dawley rat dams treated on E17 with 24 mg/kg (doses calculated as salt) intraperitoneal methylazoxymethanol acetate (MAM-E17) or saline (sham) were generated at Charles River UK, Ltd. (Kent, UK). The MAM-E17 treatment procedure was similar to that described by Moore et al. (2006). Offspring were received 12–16 weeks after birth. Rats were housed in groups of three to four per cage under a reverse (12 h:12 h) light cycle (lights off at 0700 hours) in a temperature- and humidity-controlled vivarium. After a ≥1-week acclimatization period, the animals’ access to standard diet (standard rat chow; Nestlé Purina, St. Louis, MO, USA) was restricted. Animals were maintained at ≈85–90% of their free-feeding weight throughout testing. Water was available ad libitum except during operant testing. The experiments used two cohorts of animals tested sequentially 1 year apart [cohort 1: MAM (*n* = 12) and SHAM (*n* = 7); cohort 2: MAM (*n* = 15) and SHAM (*n* = 16)]. All experiments were carried out in compliance with the UK Animals (Scientific Procedures) Act 1986 and UK Animals (Scientific Procedures) Act 1986 Amendment Regulations 2012.

### Apparatus

The experiments used 18 Med Associates, Inc. modular operant behavioral chambers (30.5 × 24.1 × 8.25 cm) modified to include a touch-sensitive monitor (Elo Touch Solutions, Inc., Milpitas, CA, USA; see Mar et al. 2013; Oomen et al. 2013) controlled through in-house software (Visual Basic 2010 Express .NET, Microsoft 2010; developed by A.C.M.). Each chamber was located within a sound-attenuating box with a fan installed for ventilation and to mask external noise. The rear wall of each chamber was equipped with a food magazine (lower center) connected to a pellet dispenser delivering 45 mg rewards, a tone generator (upper corner), and a house light (upper center). The food magazine was fitted with a tray light and a photocell beam detector for recording head entries and reward collection.

### Drugs

Drug dosing protocols were based on both in-house pilot experiments and previously published reports (Waters et al. 2005; Morgan et al. 2007; Redrobe et al. 2012; Prickaerts et al. 2012; Gastambide et al. 2012).

*Sulpiride* (Sigma-Aldrich, St. Louis, MO, USA), a dopamine D_2_/D_3_R antagonist, was mixed in sterile saline and administered intraperitoneally (i.p.) at 0, 1, 3, and 10 mg/kg doses. Sulpiride has been demonstrated to reduce certain positive and negative symptoms of schizophrenia but appears to have limited efficacy against the cognitive impairments of the disorder (Soares et al. 2000).

*Atomoxetine hydrochloride* (Sigma-Aldrich, St. Louis, MO, USA), a noradrenaline reuptake inhibitor (Wong et al. 1982), was mixed in sterile saline and injected i.p. at 0, 0.1, 0.3, and 1.0 mg/kg doses. Atomoxetine can improve certain attentional measures in individuals with ADHD (Chamberlain et al. 2007; Maziade et al. 2009; Barry et al. 2009) as well as response inhibition in healthy volunteers (Chamberlain et al. 2006).

*LSN2463359* (Eli Lilly & Co., Ltd., Windlesham, UK), a mGlu_5_R positive allo steric modulator (PAM), was suspended in 1% carboxymethyl cellulose, 0.25% Tween 80, and 0.05% antifoam and administered per os (p.o.) via syringe at 0, 1.0, 2.5, and 5.0 mg/kg doses. LSN2463359 has been demonstrated to restore cognitive inflexibility deficits in the MAM-E17 model (Gastambide et al. 2012).

*RO4938581* (F. Hoffmann La Roche, Ltd., Basel, Switzerland), an inverse agonist at the GABA(A)_α5_R, was suspended 0.3% Tween 80 and 0.05% antifoam and administered p.o. via gavage at 0, 0.1, 1.0, and 10.0 mg/kg doses. RO4938581 improves learning and memory in several rodent models (Ballard et al. 2009; Redrobe et al. 2012).

*Modafinil* (microionized; Eli Lilly & Co., Ltd., Windlesham, UK), an atypical stimulant and vigilance promoter, was suspended in 10% (*w*/*v*) sucrose and administered p.o. via syringe at 0, 8, 32, and 64 mg/kg doses. Modafinil has cognitive-enhancing and vigilance-promoting properties in various psychiatric disorders (Randall et al. 2004, 2005; Turner et al. 2004; Hunter et al. 2006; Dean et al. 2011; Schmaal et al. 2013).

*ABT-594* (AbbVie, Inc., IL, USA), a potent agonist at the α_4_β_2_nAChR (Donnelly-Roberts et al. 1998), was mixed in sterile saline and injected i.p. at 0, 1.9, 5.9, and 19 μg/kg doses. ABT-594 has been shown to improve aspects of performance on preclinical, sustained attention tasks (McGaughy et al. 1999; Mohler et al. 2010; Howe et al. 2010).

*Donepezil hydrochloride* (Sigma-Aldrich, St. Louis, MO, USA), an acetylcholinesterase inhibitor, was mixed in sterile saline and injected i.p. at 0, 0.1, 0.3, and 1.0 mg/kg doses. Donepezil can improve attentional measures in individuals with Alzheimer’s disease (Sahakian and Coull 1993; Foldi et al. 2005).

*EVP-6124* (EnVivo Pharmaceuticals, Inc., Watertown, MA, USA), a partial α_7_nAChR agonist, was suspended in 10% sucrose in deionized water vehicle and administered p.o. via syringe at 0, 0.3, 1.0, and 3.0 mg/kg doses. EVP-6124 showed some indications for therapeutic potential (Olincy et al. 2006; Freedman et al. 2008; Lieberman et al. 2013; Preskorn et al. 2014) but failed to show cognitive-enhancing effects in larger clinical trials (Fidler 2016).

### Behavioral procedure

Initial training on the rCPT was comprised of four stages. In the first stage, rats were trained to attend, approach, and touch a solid white square stimulus (7 × 7 cm) presented centrally on the touchscreen. Each white square stimulus was presented for a maximum of 10-s stimulus duration (SD). A 2-s inter-stimulus interval (ISI) was given between stimulus presentations in which only a white frame outlining the location of the response window was visible. Screen touches made within the response window either while the stimulus was being presented or in less than 500 ms following stimulus removal (SD + 500 ms = limited hold (LH) period) were designated as hits (correct responses). Following hits, the stimulus (if present) was removed immediately from the screen, the magazine light was illuminated, and a single 45 mg food reward pellet was delivered to the magazine. Reward collection extinguished the magazine light and initiated the next trial ISI. Screen touches within the response window during the ISI reset the interval, thus delaying the onset of the next stimulus presentation. Stimuli that were not touched within the LH period were classified as misses (stimulus omissions). Sessions were 45–60 min in duration. Criterion for phase 1 was defined as earning 100 rewards within a session across two consecutive sessions.

In stage 2, the white square was replaced with a novel target (S+) stimulus and the SD was reduced to 2 s (LH = 2.5 s). The new S+ was either a horizontal or vertical line stimulus, counterbalanced across both MAM and SHAM groups. Additionally, a brief ingestion delay (ID) period of 5 s was introduced following reward collection to permit the animal time to consume the food pellet before re-engaging in the task. The ISI prior to the next stimulus began immediately after termination of the ID. Animals were trained for one to two sessions on stage 2.

In stage 3, a novel nontarget (S-) stimulus was introduced to the stimulus set. The new S− was either a vertical or horizontal line stimulus—whichever was different from the S+ that was counterbalanced and randomly assigned in stage 2. The SD of both S+ and S− stimuli was equated at 2 s (LH = 2.5 s), and there was a 50% probability of either stimulus appearing on any given *normal trial*. The ISI was increased to 5 s in stage 3. Touching the S− stimulus during the LH period was designated as a false alarm (incorrect response) and resulted in immediate removal of the stimulus (if present) and initiation of a *correction-trial* ISI. On all *correction trials*, the S− was the only stimulus presented. These *correction trials* were introduced for a similar reason as the inclusion of ISI touch resetting (see stage 1—to discourage nonselective responding to stimuli and the central screen when the target stimulus is absent). Withholding responses to the S− during the LH period was designated as a correct rejection and initiated a *normal-trial* ISI. All other parameters were identical to those of stage 2. Animals were trained for seven to eight sessions on stage 3.

**Fig. 1:**
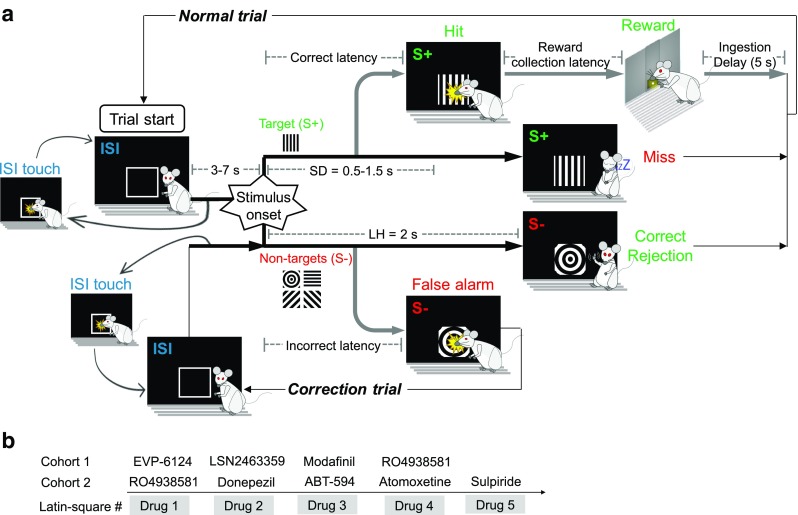
Trial structure of the rCPT and timeline of drug administration for the two cohorts of MAM-E17 and sham control rats. **a** Schematic diagram of the trial structure of the rCPT. Each session consists of a series of trials. Each trial begins (see *Trial Starts* box) with a variable inter-stimulus interval (*ISI*) = 3–7 s in which no stimuli are present. A response to the screen during the ISI is coded as an *ISI touch* and reinitiates the ISI. Following the complete ISI, either a designated target stimulus (*S+*) or one of four nontarget stimuli (*S–*) is presented individually within a central response window on a touch-sensitive monitor. Each stimulus is programmed to have a variable stimulus duration (*SD*) = 0.5–1.5 s. On *normal trials*, if an S+ is displayed, a touch to the response window during a limited hold period following stimulus onset (*LH* = 2 s) is classified as a *hit* and leads to removal of the visual stimulus and reward delivery. Following reward collection, the *normal-trial* ISI prior to the next stimulus is initiated after a brief ingestion delay (*ID*) = 5 s that affords the animal time to consume the reward. Failure to respond to the S+ stimulus within the LH is recorded as a *miss* and initiates the next *normal-trial* ISI. If an S– is displayed, omitting a response within the LH is classified as a *correct rejection* and initiates the next *normal-trial* ISI. A response-window touch during the LH of S– presentation is classified as a *false alarm* and causes removal of the visual stimulus and onset of a *correction*-trial ISI. *Correction trials*—in which a randomly selected S– is presented—are repeated until a successful *correct rejection* is completed. **b** Timeline illustrating the order of the drug administration studies in cohorts 1 and 2 of MAM-E17 and sham control rats

A flowchart overview of the rCPT trial structure for the fourth and final training stage is depicted in Fig. 1a. In this stage, three additional S– stimuli were introduced to the stimulus set. All stimuli were luminance matched in an attempt to equate their salience for simple brightness detection. The SD of S+ and S– stimuli was set to be variable (0.5–1.5 s) but with a fixed LH of 2 s. The LH remained at 2 s after stimulus onset to maintain the average event rate and to ensure that animals had equal opportunity to respond to each stimulus. As with stage 3, the probability of presentation of either an S+ or an S– stimulus on normal trials was equal (50%). The ISI was also set to be variable (3–7 s). The variable SD and ISI were introduced to increase the attentional load of the task. Other parameters were the same as stage 3, with touches to any of the S– stimuli resulting in a correction trial in which an S– was presented randomly from the set of four. All animals were trained a minimum of 20 sessions on stage 4 to establish reliable rCPT performance prior to drug testing. Animals were trained on the rCPT for daily sessions, 5–7 days a week.

### Experimental design: drug testing

The effects of different compounds were investigated using stage 4 performance parameters. Drugs were administered following a diagram-balanced Latin square design (Cardinal and Aitken 2006). Each drug Latin square experiment consisted of four test sessions with one dose per session. Each dosing session was separated by at least 48 h with no-drug *baseline* sessions run in between. A typical minimum interval of 1 week, with several no-drug baseline retention sessions being run, was given between different drug treatments. The order of the drugs administered to the two cohorts is illustrated in Fig. 1b. In cohort 1, the effects of EVP-6124, LSN2463359, modafinil, and RO4938581 were examined on rCPT performance. The session duration was 60 min in cohort 1 but was restricted to 45 min in cohort 2 as it had been determined that the additional 15 min did not greatly alter the pattern of rCPT results. In cohort 2, RO4938581, donepezil, ABT-594, atomoxetine, and sulpiride were tested. RO4938581 was assessed in both cohorts to control for cross-batch reproducibility of pharmacological effects. Prior to the Latin square experiment for ABT-594, a preliminary dose (19 μg/kg) was administered in a crossover design during rCPT testing to permit acclimation to the initial effects of the drug (Mohler et al. 2010). All compounds were administered 30 min prior to rCPT testing.

### Data analysis

Only the first 45 min of each session was analyzed to better compare data across cohorts. However, no significant differences in performance variables were observed regardless of whether a 45- or 60- min session was analyzed in cohort 1.

#### Behavioral parameters

Analogous to human CPTs, a response to the target (S+) was classified as a *hit*, failure to respond to the target was classified as a *miss*, withholding from responding to a nontarget (S–) was recorded as a *correct rejection* (CR), and responding to a nontarget was classified as a *false alarm*. Responses to the screen during the ISI (when no stimulus was present) were coded as *ISI touches*. For each animal, *hit rate* (HR) was calculated as the number of hits as a proportion of the total number of CS+ presentations (hits / (hits + misses). *False alarm rate* (FAR) was calculated as the number of false alarms as a proportion of the total number of CS– presentations (FA / (FA + CR)). The discrimination sensitivity index was calculated as (Macmillan and Creelman 2004)

$$ {d}^{\prime }=z\left(\mathrm{hit}\kern0.5em \mathrm{rate}\right)-z\left(\mathrm{false}\kern0.5em \mathrm{alarm}\kern0.5em \mathrm{rate}\right) $$

with higher values indicating better discrimination sensitivity across the session. The response criterion was calculated as

$$ c=-0.5\left(z\left(\mathrm{hit}\kern0.5em \mathrm{rate}\right)+z\left(\mathrm{false}\kern0.5em \mathrm{alarm}\kern0.5em \mathrm{rate}\right)\right) $$

with larger values indicating decreased overall responding to both the target and nontarget stimuli. An identical pattern of significant effects was obtained when nonparametric indices of sensitivity and response bias were calculated (Stanislaw and Todorov 1999), which do not depend on assumptions such as normal distributions and equal variances for the discriminability of targets and nontargets. The primary dependent variables were sensitivity index (*d*′), response criterion (*c*), hit rate, false alarm rate, and ISI touches. Correct response latency, incorrect response latency, and reward retrieval latency were also assessed.

Independent sample *t* tests were used to compare MAM and sham performance in stage 1 (sessions to criterion), stage 2 (average hit rate), and stage 3 (*d*′). To assess the robustness of stage 4 rCPT performance in MAM and sham control animals, the average performance of two to three drug-free baseline sessions for each measure was analyzed at time points immediately prior to each Latin square experiment. Only four of five of the equivalent time points were analyzed in cohort 2 for comparison with cohort 1. A three-way mixed ANOVA was used in which *cohort* [1, 2] and *group* [MAM, sham] were included as between-subjects factors and *time point* [1, 2, 3, 4] was included as a within-subjects factor. To further examine the stability of the MAM versus sham differences, each cohort was also analyzed separately at each time point from cohorts 1 and 2 with *group* as a factor. Effect sizes for the difference in *d*′ between MAM and sham rats at each time point were also calculated. The vehicle treatment trials from each Latin square experiment were also analyzed in a similar manner.

Data from each drug experiment were analyzed using repeated measures ANOVA, with *group* (between-subjects, two levels of dose: sham versus MAM treatment) and *drug dose* (within-subjects, four levels of dose) as independent variables. Significant main effects of drug were followed by post hoc comparisons against vehicle using the Šidák adjustment for multiple comparisons. Significant interactions were investigated further by analysis of simple main effects and Šidák-corrected pairwise comparisons. Data were analyzed using SPSS Statistics 21 (IBM Corp., Armonk, NY, USA). We additionally confirmed all of our repeated measures ANOVA results using an alternate statistical model (replicated Latin square design with a between-subjects factor) that accounted for day and order effects (Cardinal and Aitken 2006). Moreover, we found no significant differences in our results when correction trials were either included or excluded from the analysis.

## Results

### Effect of MAM-E17 on rCPT performance

**Fig. 2:**
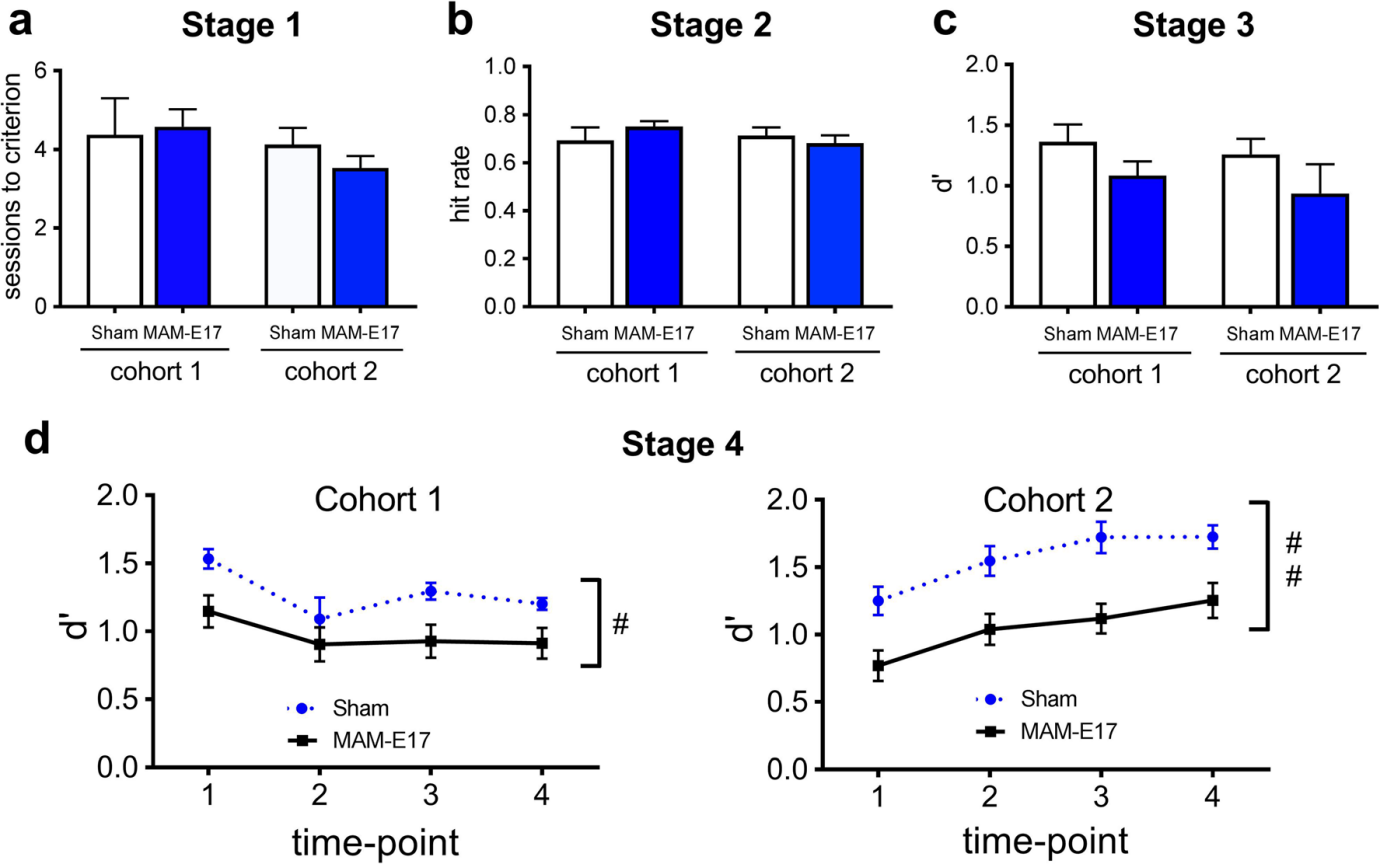
rCPT acquisition data and long-term stability of *baseline* task performance in MAM-E17 and sham control animals. **a** *Stage 1* no differences between MAM-E17 and sham groups on the number of sessions to criterion. **b** *Stage 2* no differences between MAM-E17 and sham groups on mean hit rate. **c** *Stage 3* no differences between MAM-E17 and sham animals on performance sensitivity, *d*′, averaged across the last 3 days of testing. **d** *Stage 4* mean *d*′ in the rCPT at four baseline time points for cohorts 1 and 2. Each time point comprises two to three sessions prior to commencing a Latin square drug study. MAM-E17 rats show stable deficits in *d*′ across the four time points spanning several months. There were no significant effects of cohort or cohort × group interactions. Data are expressed as mean ± SEM. *Hash tags* denote significant main effects of group (^#^ *p* < 0.05; ^##^ *p* < 0.01)

Across the two cohorts of animals and eight drug studies spanning several months, the MAM-E17 rats showed several highly consistent and selective differences in rCPT performance relative to sham controls (Fig. 2d, Table 1). During rCPT acquisition, there were no significant differences between MAM and sham control rats or between cohorts 1 and 2 in terms of the number of sessions to criterion in stage 1 (Fig. 2a), the hit rate or number of pellets earned per session during stage 2 (Fig. 2b), or the sensitivity (*d*′) during stage 3 (Fig. 2c). However, when examining stage 4 rCPT performance across baseline sessions prior to each Latin square drug study, MAM animals exhibited significantly decreased *d*′ (Fig. 2d; *F*[1,46] = 15.815, *p* < 0.001) and significantly increased false alarm rate (*F*[1,46] = 18.160, *p* < 0.001) and number of ISI screen touches (*F*[1,46] = 7.030, *p* = 0.011) relative to sham controls in both cohorts 1 and 2. There were no significant main effects of cohort or group × cohort interactions for any of the measures, suggesting that the MAM-E17 model induced specific and robust performance differences in the rCPT relative to sham controls. When each cohort and time point was analyzed separately, the effects on both *d*′ and false alarm rate were significant at three of four time points in cohort 1 (all *p* values <0.05) and at every time point in cohort 2 (all *p* values <0.05), highlighting the stability of the observed differences between MAM-E17 and sham rats across many months. The effect sizes of the differences in *d*′ between MAM-E17 and shams at each time point were large, ranging from 0.68 to 1.34 in cohort 1 (overall = 1.23) and from 1.10 to 1.37 in cohort 2 (overall = 1.27).

**Table 1:** Summary of the MAM-E17 rat model phenotype relative to the sham controls on rCPT performance variables

Performance measure	MAM-E17 phenotype
Hit rate	–
False alarm rate	↑
*d*′	↓
*c*	–
ISI touches	↑
Correct latency	–
Incorrect latency	–
Retrieval latency	–

↑ = increased in the MAM-E17 model; ↓ = decreased in the MAM-E17 model; – = not affected in the MAM-E17 model

A similar pattern was observed when measures were averaged across only the vehicle treatment sessions in both cohorts (four and five sessions in cohorts 1 and 2, respectively). MAM-E17 rats showed significantly lower performance sensitivity, *d*′, relative to sham control animals (*F*[1,46] = 13.480, *p* = 0.001). In addition, MAM-E17 rats displayed significantly higher FAR (*F*[1,46] = 13.189, *p* = 0.001) and ISI touches (*F*[1,46] = 7.746, *p* = 0.008). There were no MAM-E17 effects on any other measures (HR, *c*, as well as correct, incorrect, and reward collection latencies). There were no significant main effects of cohort or group × cohort interactions for any of the measures, again suggesting that the observed effects of the MAM-E17 model on rCPT performance were specific, stable, and reproducible.

### Effect of acute drug treatments on rCPT performance

**Fig. 3:**
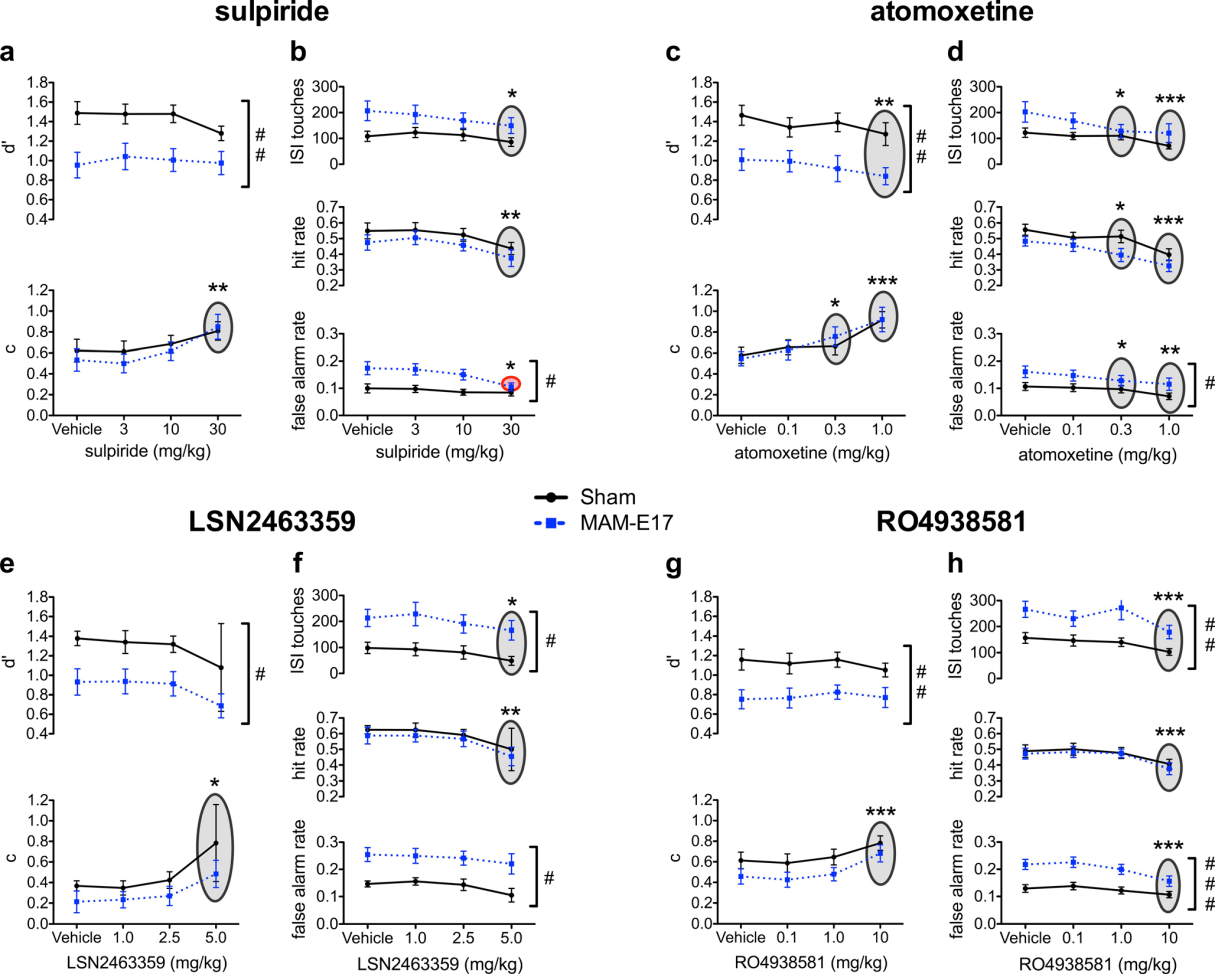
Effect of sulpiride, atomoxetine, LSN2463359, and RO4938581 on rCPT performance in MAM-E17 and sham control rats. All significant group differences are relative to sham controls, and significant drug effects are in comparison to the vehicle treatment. For all compounds, MAM-E17 rats exhibited a dose-independent decrease in *d*′ relative to shams (**a**, **c**, **e**, **g**) **a**, **b** Sulpiride. **a** Sulpiride (30 mg/kg) increased response criterion (*c*) in MAM-E17 animals without affecting *d*′. **b** Sulpiride (30 mg/kg) reduced the elevated FAR selectively in MAM-E17 animals and caused reductions in HR and ISI touches across both MAM-E17 and sham animals. **c**, **d** Atomoxetine. **c** Atomoxetine increased *c* at both 0.3 and 1.0 mg/kg doses and reduced *d*′ at a 1.0 mg/kg dose. **d** Atomoxetine decreased HR and *d*′ at 0.3 and 1.0 mg/kg and reduced FAR at 1.0 mg/kg. MAM-E17 animals showed a dose-independent increase in FAR relative to shams. **e**, **f** LSN2463359. **e** LSN2463359 (5 mg/kg) increased *c* across both groups of animals. MAM-E17 animals showed a dose-independent decrease in *d*′ compared to shams. **f** LSN2463359 (5 mg/kg) reduced HR and ISI touches in both MAM-E17 and sham groups. MAM-E17 animals showed a dose-independent increase in FAR. **g**, **h** RO4938581. **g** RO4938581 (10 mg/kg) increased *c* in both groups of rats. MAM-E17 animals showed a dose-independent decrease in *d*′ relative to sham controls. **h** RO4938581 (10 mg/kg) decreased HR, FAR, and ISI touches in both groups. MAM-E17 animals showed a dose-independent increase in FAR compared to sham controls. *Gray shading with asterisks* denotes the main effect of drug dose with a significant difference from vehicle. *Red shading with asterisks* denotes group × dose interaction with a significant difference from vehicle (*p < 0.05; **p < 0.01; ***p < 0.0001). *Hash tags* denote significant main effects of group (^#^ *p* < 0.05; ^##^ *p* < 0.01; ^###^ *p* < 0.0001)

**Fig. 4:**
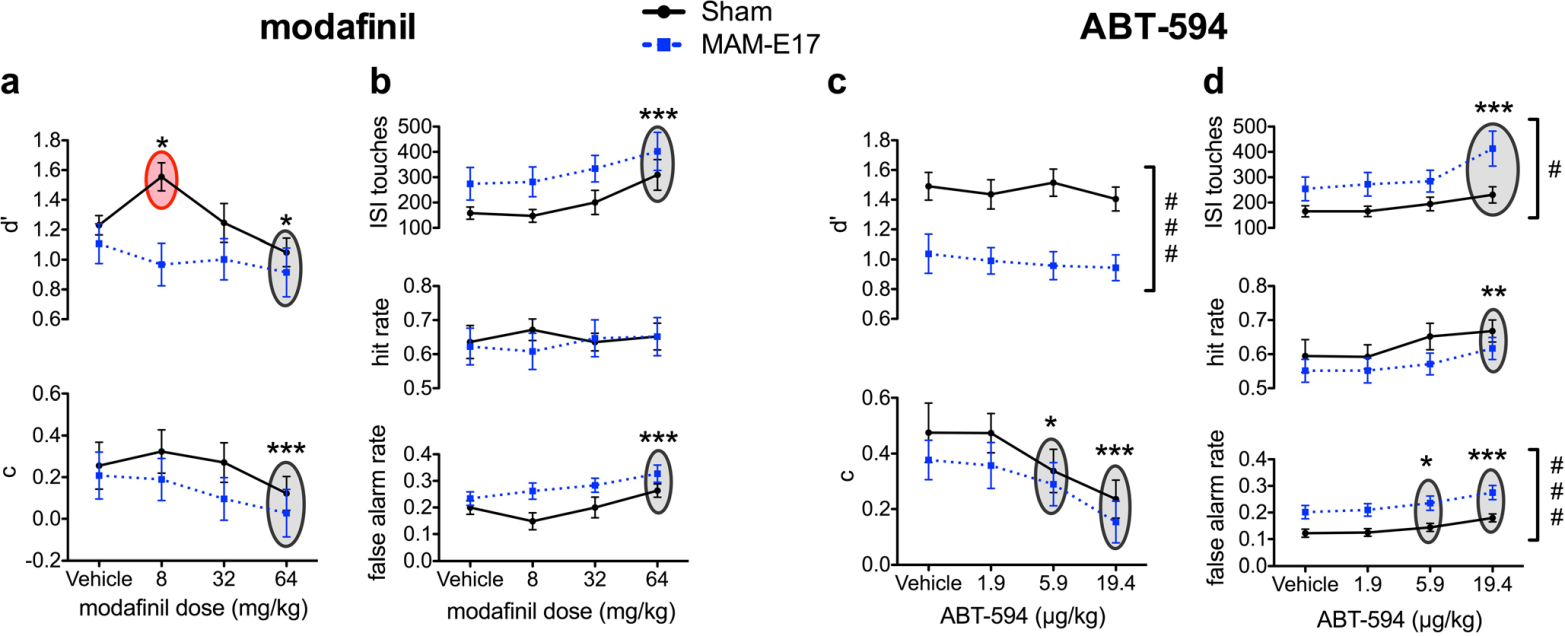
Effect of modafinil and ABT-594 on rCPT performance in sham and MAM-E17-treated rats. All significant group differences are relative to sham controls, and significant drug effects are in comparison to the vehicle treatment. **a**, **b**. Modafinil. **a** At 8 mg/kg, modafinil increased *d*′ selectively in the sham group without affecting *d*′ in MAM-E17 animals. At 64 mg/kg, modafinil decreased *d*′ and response criterion (*c*) across both groups. **b** At 64 mg/kg, modafinil increased FAR and ISI touches and in both groups. **c**, **d** ABT-594. **c** ABT-594 caused group-independent decreases in *c* at both 5.9 and 19.4 μg/kg doses. MAM-E17 animals showed dose-independent reductions in *d*′ relative to controls. **d** ABT-594 (19.4 μg/kg) also increased FAR, HR, and ISI touches in both groups. MAM animals showed dose-independent increases in FAR and ISI touches. *Gray shading with asterisks* denotes the main effect of drug dose with a significant difference from vehicle. *Red shading with asterisks* denotes group × dose interaction with a significant difference from vehicle (*p < 0.05; **p < 0.01; ***p < 0.0001). *Hash tags* denote significant main effects of group (^#^ *p* < 0.05; ^###^ *p* < 0.0001)

**Fig. 5:**
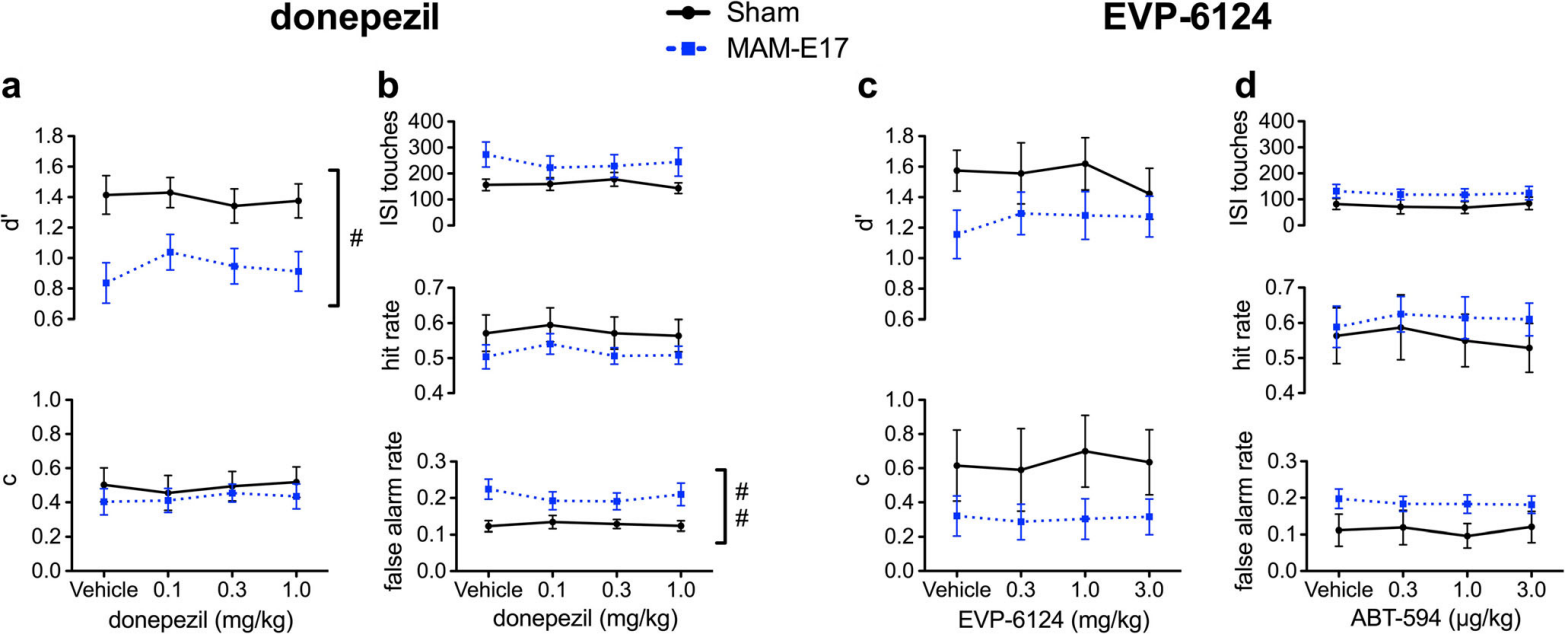
Effect of donepezil and EVP-6124 on rCPT performance in sham and MAM-E17-treated rats. **a**, **b** Donepezil. **a** There was a near-significant improvement in *d*′ by donepezil (0.1 mg/kg) across both groups of animals. MAM-E17 animals exhibited dose-independent decreases in *d*′. **b** No effects of EVP-6124 on HR, FAR, or ISI touches. MAM-E17 animals showed a dose-independent increase in FAR. **c**, **d**. EVP-6124. **c**, **d** No effects of EVP-6124 on any principal rCPT performance indices. *Gray-shaded asterisk* denotes the main effect of drug dose with a significant difference from vehicle. *Hash tags* denote a significant main effect of group (^#^ *p* < 0.05; ^##^ *p* < 0.01)

All of the drug effects on the primary rCPT performance indices (*d*′, HR, FAR, *c*, and ISI touches) are presented in Figs. 3, 4, and 5 and summarized in Table 2 and Supplementary Table S1. Latency measures (correct response, incorrect response, and reward collection latency) are presented in Table 3. Only significant and near-significant effects are highlighted below. Results on nonsignificant measures are included in the Supplement.

**Table 2:** Summary of pharmacological effects on the main rCPT performance variables

Drug	Hit rate	False alarm rate	*d*′	*c*	ISI touches
Sulpiride	↓	↓^a^	–	↑	↓
Atomoxetine	↓	↓	↓	↑	↓
LSN2463359	↓	\	–	↑	↓
RO4938581	↓	↓	–	↑	↓
Modafinil	–	↑	↑ (low dose)^b^; ↓ (high dose)	↓	↑
ABT-594	↑	↑	–	↓	↑
Donepezil	–	–	/	–	–
EVP-6124	–	–	–	–	–

↓ = significant decrease; ↑ = significant increase; \ = trend toward decrease; / = trend toward increase; – = no effect

^a^Effect in the MAM-E17 model only

^b^Effect in the sham controls only

**Table 3:**
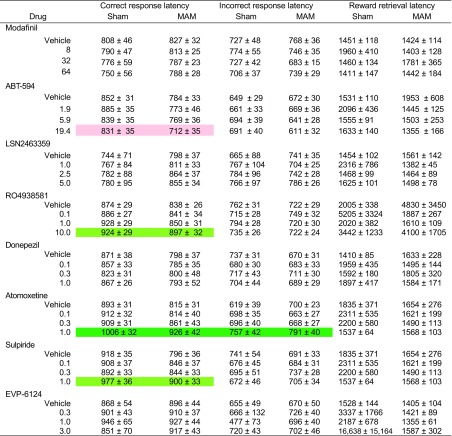
Response and retrieval latencies of MAM-E17 and sham animals in the rCPT during acute systemic pharmacological challenges

For the significant effects of the drug dose denoted by color with statistical significance highlighted by color code, please see the legend

#### Sulpiride

Sulpiride (0–30 mg/kg) dose-dependently and selectively reduced the elevated FAR of MAM animals on the rCPT (Fig. 3b, Table 2). It also dose-dependently lowered overall levels of responding (increased *c* with decreased HR, FAR, and ISI touches) and increased correct response latency (Fig. 3a, b, Table 3). One sham animal was excluded from analysis due to a data acquisition error during the vehicle dose session.

For FAR, there was both a significant main effect of dose (dose: *F*[3,84] = 10.203, *p* < 0.001; H-F corrected) and a significant dose × group interaction (dose × group: *F*[3,84] = 4.119, *p* = 0.016; H-F corrected). Post hoc tests confirmed that sulpiride significantly reduced FAR selectively in MAM-E17 animals at the highest 30 mg/kg dose relative to vehicle (*p* < 0.05), such that MAM-E17 animals no longer displayed significantly elevated FAR relative to sham controls at the 30 mg/kg dose (*p* = 0.206 where *p* < 0.05 at all other sulpiride doses). There were no significant FAR changes in sham control animals under sulpiride treatment. There were also significant main effects of sulpiride dose on HR (dose: *F*[3,84] = 7.614, *p* < 0.001; dose × group: *F*[3,84] = 0.149, *p* = 0.900; H-F corrected), *c* (dose: *F*[3,84] = 10.921, *p* < 0.001; dose × group: *F*[3,84] = 0.994, *p* = 0.385; H-F corrected), and ISI touches (dose: *F*[3,84] = 3.977, *p* < 0.014; dose × group: *F*[3,84] = 0.867, *p* = 0.450; H-F corrected). Within-subjects contrasts revealed significant linear relationships between ascending sulpiride doses and reductions in HR (*F*[1,28] = 9.623, *p* = 0.004) and ISI touches (*F*[1,28] = 7.947, *p* = 0.009), as well as with increases in *c* (*F*[1,28] = 15.014, *p* < 0.001). Post hoc tests showed that the highest 30 mg/kg sulpiride dose significantly reduced HR and ISI touches and significantly increased response criterion, *c*, relative to vehicle-treated animals across both MAM and sham control groups (all *p* < 0.05).

There was a significant main effect of sulpiride on correct response latency (dose: *F*[3,84] = 8.145, *p* < 0.001; dose × group: *F*[3,84] = 1.610, *p* = 0.193), with linear dose-dependent increases (*F*[1,29] = 12.451, *p* = 0.001) such that the highest 30 mg/kg sulpiride dose resulted in longer correct response latencies relative to the vehicle treatment (*p* < 0.05).

#### Atomoxetine

Atomoxetine (0–1 mg/kg) had significant suppressant effects on all of the primary response measures (*d*′, *c*, HR, FAR, ISI touches) and increased correct and incorrect response latencies in the rCPT (Fig. 3c, d, Tables 2 and 3).

Atomoxetine caused dose-dependent decreases in performance sensitivity, *d*′ (dose: *F*[3,87] = 2.896, *p* = 0.040; dose × group: *F*[3,87] = 0.408, *p* = 0.748), HR (dose: *F*[3,87] = 11.517, *p* < 0.001; dose × group: *F*[3,87] = 0.533, *p* = 0.661), FAR (dose: *F*[3,87] = 6.796, *p* < 0.001; dose × group: *F*[3,87] = 0.483, *p* = 0.695), and ISI touches (dose: *F*[3,87] = 8.729, *p* < 0.001; dose × group: *F*[3,87] = 1.830, *p* = 0.148). It also significantly increased response criterion, *c* (dose: *F*[3,87] = 12.473, *p* < 0.001; dose × group: *F*[3,87] = 0.462, *p* = 0.709). Within-subjects contrasts revealed significant linear effects on all of these measures, showing that increasing atomoxetine doses were associated with decreases in *d*′ (*F*[1,29] = 9.033, *p* = 0.005), HR (*F*[1,29] = 23.855, *p* < 0.001), FAR (*F*[1,29] = 12.977, *p* = 0.001), and ISI touches (*F*[1,29] = 29.132, *p* < 0.001), as well as with increases in *c* (*F*[1,29] = 22.471, *p* < 0.001). Post hoc tests showed that, relative to the vehicle treatment, the highest 1 mg/kg dose significantly decreased *d*′, HR, and FAR and that both 0.3 and 1 mg/kg doses led to significantly fewer ISI touches and an increased response criterion, *c* (all *p* values <0.05).

There were significant main effects of atomoxetine dose on both correct response latency (dose: *F*[3,87] = 12.295, *p* < 0.001; dose × group: *F*[3,87] = 0.517, *p* = 0.672) and incorrect response latency (dose: *F*[3,87] = 8.692, *p* < 0.001; dose × group: *F*[3,87] = 2.512, *p* = 0.075; H-F corrected). Within-subjects contrasts revealed significant linear effects indicating a dose-dependent slowing of correct (*F*[1,29] = 22.472, *p* < 0.001) and incorrect (*F*[1,29] = 13.772, *p* < 0.001) response latencies. Post hoc comparisons showed that the highest 1 mg/kg dose of atomoxetine resulted in prolonged response latencies relative to the vehicle treatment (all *p* values <0.05).

#### LSN2463359

LSN2463359 (0–5 mg/kg) dose-dependently reduced levels of rCPT responding, as evidenced by significant changes in HR, *c*, and ISI touch parameters across both MAM-E17 and sham control groups (Fig. 3e, f, Tables 2 and 3). Two sham animals did not receive one of the appropriate LSN2463359 dose levels and were thus excluded from statistical analysis.

There were significant main effects of LSN2463359 dose on HR (dose: *F*[3,45] = 4.893, *p* = 0.017; dose × group: *F*[3,45] = 0.023, *p* = 0.970; H-F corrected), *c* (dose: *F*[3,45] = 5.729, *p* = 0.016; dose × group: *F*[3,45] = 0.357, *p* = 0.638; H-F corrected), and ISI touches (dose: *F*[3,45] = 3.681, *p* = 0.019; dose × group: *F*[3,45] = 0.197, *p* = 0.898). Within-subjects contrasts revealed significant patterns such that increasing LSN2463359 doses were linearly associated with decreases in HR (*F*[1,15] = 8.291, *p* = 0.011) and ISI touches (*F*[1,15] = 6.921, *p* = 0.019), as well as with linear increases in *c* (*F*[1,15] = 7.919, *p* = 0.013). Post hoc comparisons showed that the highest 5 mg/kg LSN2463359 dose significantly reduced HR and ISI touches and increased response criterion, *c*, relative to vehicle-treated animals across both MAM-E17 and sham control groups (all *p* values <0.05). There was also a nonsignificant trend for higher LSN2463359 doses to reduce FAR (dose: *F*[3,45] = 2.395, *p* = 0.081; dose × group: *F*[3,45] = 0.159, *p* = 0.923).

There was a significant dose × group interaction for reward collection latency (dose: *F*[3,42] = 2.110, *p* = 0.144; dose × group: *F*[3,42] = 3.609, *p* = 0.045; H-F corrected), but post hoc tests revealed no significant pairwise comparisons.

#### RO4938581

RO4938581 (0–10 mg/kg) dose-dependently reduced overall levels of responding on the rCPT, as indicated by an increased *c* parameter; decreased HR, FAR, and ISI touches; and increased correct response latencies (Fig. 3g, h, Tables 2 and 3). The effects were highly consistent across both cohorts of animals. From both cohorts 1 and 2, four of 27 MAM-E17 rats and one of 23 sham control animals had data missing from one of the dosing sessions and were thus excluded from statistical analysis.

There were significant main effects of RO4938581 dose on HR (dose: *F*[3,129] = 7.553, *p* < 0.001; dose × group: *F*[3,129] = 0.011, *p* = 0.998), FAR (dose: *F*[3,129] = 10.134, *p* < 0.001; dose × group: *F*[3,129] = 1.570, *p* = 0.200), *c* (dose: *F*[3,126] = 9.395, *p* < 0.001; dose × group: *F*[3,126] = 0.053, *p* = 0.984), and ISI touches (dose: *F*[3,129] = 7.172, *p* < 0.001; dose × group: *F*[3,129] = 0.845, *p* = 0.457; H-F corrected). Within-subjects contrasts revealed significant linear patterns, with increasing RO4938581 doses associated with reductions in HR (*F*[1,43] = 11.533, *p* = 0.001), FAR (*F*[1,43] = 28.607, *p* < 0.001), and ISI touches (*F*[1,43] = 23.687, *p* < 0.001), as well as with increases in *c* (*F*[1,42] = 19.849, *p* < 0.001). Post hoc tests showed that, compared to the vehicle treatment, the highest 10 mg/kg RO4938581 dose significantly reduced HR, FAR, and the number of ISI touches and increased response criterion, *c*, across both MAM-E17 and sham control groups (all *p* values <0.001).

There was a significant main effect of RO4938581 dose on correct response latency (dose: *F*[3,126] = 4.580, *p* = 0.004; dose × group: *F*[3,126] = 0.358, *p* = 0.783), with a significant within-subjects contrast indicating that ascending doses were linearly related with increases in correct response latencies (*F*[1,42] = 15.055, *p* < 0.001). Post hoc comparisons showed that the 10 mg/kg RO4938581 dose significantly increased correct latencies relative to the vehicle treatment (*p* values <0.05).

To examine the consistency of RO4938581 effects across cohorts, the data were analyzed adding cohort as a fixed factor. Across all dependent measures, there was only a single significant effect of cohort: a group × dose × cohort interaction on the response criterion index, *c* (*F*[3,120] = 3.791, *p* = 0.012), in which sham control animals from cohort 1 had a decreased *c* parameter when they were administered 1 mg/kg RO4938581 as compared to similarly treated animals from cohort 2 (*p* < 0.05). Apart from this isolated effect, there were no significant main effects of cohort or interactions involving cohort × group, cohort × dose, or cohort × group × dose for any of the rCPT performance measures. The general absence of any statistical effects of cohort on the main findings of RO4938581 underscores the reliability and reproducibility of these drug effects on the rCPT paradigm.

#### Modafinil

Modafinil (8 mg/kg) selectively improved performance sensitivity, *d*′, in sham control rats (Fig. 4a, Table 2). Modafinil (0–64 mg/kg) also dose-dependently increased levels of responding within the rCPT, as demonstrated by decreases in *c*, increases in FAR and ISI touches, and linear reductions in correct response latency with increasing modafinil dose (Fig. 4a, b, Tables 2 and 3).

For performance sensitivity, *d*′, there was both a significant main effect of dose (*F*[3,51] = 4.396, *p* = 0.008) and a significant dose × group interaction (*F*[3,51] = 3.834, *p* = 0.015). Post hoc tests confirmed that low-dose (8 mg/kg) modafinil selectively enhanced *d*′ in sham control animals (increased *d*′ relative to sham vehicle, *p* < 0.05). Post hoc comparisons within the main effect of dose also showed that, relative to vehicle, the highest 64 mg/kg modafinil dose reduced *d*′ performance across both MAM-E17 and sham control animals (*p* < 0.05). There were significant main effects of modafinil dose on FAR (dose: *F*[3,51] = 12.304, *p* < 0.001; dose × group: *F*[3,51] = 2.152, *p* = 0.105), *c* (dose: *F*[3,51] = 6.511, *p* = 0.001; dose × group: *F*[3,51] = 0.747, *p* = 0.529), and ISI touches (dose: *F*[3,51] = 8.480, *p* < 0.001; dose × group: *F*[3,51] = 0.184, *p* = 0.907). Within-subjects contrasts revealed significant linear relationships, with an increasing modafinil dose associated with increases in FAR (*F*[1,17] = 35.048, *p* < 0.001) and ISI touches (*F*[1,17] = 17.681, *p* < 0.001), as well as a decreases in response criterion, *c* (*F*[1,17] = 20.678, *p* < 0.001). Post hoc tests showed that, relative to the vehicle treatment, the highest 64 mg/kg modafinil dose significantly elevated the FAR and number of ISI touches and decreased *c* across all animals (all *p* values <0.05).

Within-subjects trend analysis also revealed a significant linear relationship in which higher modafinil doses were associated with faster correct response latencies (*F*[1,17] = 8.367, *p* < 0.010).

#### ABT-594

ABT-594 (0–19.4 μg/kg) dose-dependently increased overall levels of responding by decreasing the *c* parameter and increasing HR, FAR, and ISI touches. ABT-594 also dose-dependently decreased correct response latencies (Fig. 4c, d, Tables 2 and 3).

In the acclimation to ABT-594 dosing using a crossover design (data not shown), there was a significant group × dose interaction (*F*[1,29] = 4.932, *p* < 0.05) observed for ISI touches, in which 19.4 μg/kg ABT-594 increased ISI touches selectively in MAM rats.

In the Latin square, there were significant main effects of ABT-594 dose on HR (dose: *F*[3,87] = 5.897, *p* = 0.001; dose × group: *F*[3,87] = 0.422, *p* = 0.724), FAR (dose: *F*[3,87] = 17.807, *p* < 0.001; dose × group: *F*[3,87] = 0.238, *p* = 0.870), *c* (dose: *F*[3,87] = 15.072, *p* < 0.001; dose × group: *F*[3,87] = 0.276, *p* = 0.842), and ISI touches (dose: *F*[3,87] = 13.432, *p* < 0.001; dose × group: *F*[3,87] = 2.552, *p* = 0.083; H-F corrected). Within-subjects contrasts revealed significant linear increases in HR (*F*[1,29] = 13.385, *p* = 0.001), FAR (*F*[1,29] = 42.314, *p* < 0.001), and ISI touches (*F*[1,29] = 26.450, *p* < 0.001), as well as a significant linear decrease in *c* (*F*[1,29] = 32.801, *p* < 0.001) in response to increasing ABT-594 doses. Post hoc comparisons showed that the highest 19.4 μg/kg ABT-594 dose significantly increased HR, FAR, and ISI touches, and both the 5.9 and 19.4 μg/kg doses decreased response criterion (*c*) relative to vehicle-treated animals (all *p* values <0.05).

ABT-594 dose-dependently speeded correct response latencies (dose: *F*[3,87] = 4.698, *p* = 0.004; dose × group: *F*[3,87] = 1.362, *p* = 0.260) where within-subjects contrasts revealed a linear decrease in latencies across ascending doses (*F*[1,29] = 8.606, *p* = 0.006). Post hoc tests showed that the highest 19.4 μg/kg dose decreased correct response latencies compared to the vehicle treatment (*p* < 0.05). There was a significant dose × group interaction on incorrect response latency (dose: *F*[3,87] = 0.309, *p* = 0.819; dose × group: *F*[3,87] = 3.356, *p* = 0.022), but post hoc tests revealed no significant pairwise comparisons.

#### Donepezil

There was a strong but nonsignificant trend for donepezil (0–1 mg/kg) to improve performance sensitivity, *d*′, with no impact on any other rCPT measures (Fig. 5a, b, Tables 2 and 3). One MAM-E17 and one sham rat had missing data due to computer hardware failure during testing and were excluded from statistical analysis. There was a trend for a main effect of donepezil to enhance *d*′ performance across both MAM-E17 and sham control rats (dose: *F*[3,81] = 2.584, *p* = 0.064; dose × group: *F*[3,81] = 1.256, *p* < 0.295; H-F corrected). This trend toward performance enhancement was most pronounced at the lowest 0.1 mg/kg donepezil dose relative to the vehicle treatment. There were no significant effects of donepezil on any other performance measure in the rCPT.

#### EVP-6124

EVP-6124 (0–3 mg/kg) did not alter any performance measure in the rCPT (Fig. 5c, d, Tables 2 and 3). One MAM-E17 animal made no responses at the 0.3 mg/kg dose and was thus necessarily excluded from statistical analyses of *d*′, *c*, and response latencies. An identical pattern of statistical results was obtained if the animal was excluded from all measures.

There was a near-threshold significant main effect of EVP-6124 dose on correct response latency (dose: *F*[3,48] = 2.776, *p* = 0.050; dose × group: *F*[3,48] = 1.346, *p* = 0.270), where the largest effect was a slower correct response time at the 1 mg/kg dose.

## Discussion

### MAM-E17 model impairments on rCPT

MAM-E17 rats showed robust and persistent deficits in discriminative sensitivity (*d*′) on the rCPT relative to sham controls. The effect size was large, virtually identical in two cohorts of animals (Cohen’s *d* = 1.23 and 1.27), and consistent across repeated drug testing. This model profile is remarkably similar to impairments on common variants of human CPTs widely reported in neuropsychiatric disorders such as ADHD or schizophrenia (Chen and Faraone 2000; Riccio et al. 2002). For example, a meta-analysis examining CPT performance across 15 studies reported substantial *d*′ impairments (*d* = 1.18) in schizophrenia compared to control subjects (Heinrichs and Zakzanis 1998). A large, multi-site study found similar *d*′ decrements in schizophrenia on three-digit (*d* = 1.13) and four-digit (*d* = 1.14) identical-pairs CPT (Nuechterlein et al. 2015). Moreover, several studies have demonstrated that such *d*′ deficits may remain stable over time irrespective of psychotic/remitted state, positive or negative symptoms or medication status, and are reliable across repeated testing (Cornblatt et al. 1997; Nuechterlein et al. 2015).

The MAM-E17 deficits in *d*′ were observed in the absence of significant changes in response bias/criterion (*c*). This dissociation between *d*′ and *c* mirrors what is commonly reported in schizophrenia research using CPT (Mussgay and Hertwig 1990; Nuechterlein et al. 2015). Response bias/criterion is theoretically independent of *d*′ and defines the extent to which a particular response type or strategy (e.g., to respond or withhold responding) is preferred or more probable (Stanislaw and Todorov 1999; Macmillan and Creelman 2004). In line with interpretations of the human CPT literature, the robust and selective reduction in *d*′ for the MAM-E17 model may be attributable to impairments in sustained, focused, and/or selective attentional processes—the ability to maintain readiness and to detect, discriminate, and respond appropriately to rapidly and successively presented target stimuli across time (Parasuraman 1979; Davies and Parasuraman 1982).

Schizophrenia-related deficits in *d*′ are frequently reported in conjunction with decreases in the number or rate of detected target stimuli (Cornblatt et al. 1989; Nuechterlein et al. 2015). In the current study, MAM-E17 rats displayed a consistent, but nonsignificant, decrement in target HR relative to sham controls. The discrepancy in effect sizes for HR between rodent and human versions might reflect differences in cost/benefit structure. In the rCPT, there is a high incentive for animals to maximize target hits (immediate food rewards) with modest punishment (delay) for occasional errant responses. MAM-E17 rats might thus compensate for a lower *d*′ by slightly adjusting their response strategy (e.g., higher FAR) to maximize rewards (Lynn and Barrett 2014). By contrast, human CPT variants typically offer no immediate incentives to encourage target hits (Locke and Braver 2008). It would be interesting to examine how systematic alterations in the cost/benefit matrix of the rCPT or human CPTs alter the performance of the MAM-E17 model and individuals with schizophrenia, respectively.

The MAM-E17 model also has a profile consistent with deficits in inhibitory control on the rCPT, showing significant increases in nontarget FAR and in the number of screen touches in the absence of any stimuli (ISI touches). General locomotor hyperactivity of MAM-E17 animals has been reported in several studies (Le Pen et al. 2006; Hazane et al. 2009; Ratajczak et al. 2015; O’Reilly et al. 2016; but see Flagstad et al. 2004) and discussed as a possible confound in the assessment of cognitive function (O’Reilly et al. 2016). However, it is unlikely that excessive, nonspecific motor activity of MAM-E17 rats is a large contributing factor to cognitive performance in the rCPT as we observed no significant group differences on HR, overall response bias/criterion, or any reaction time latencies. More specific impairments in inhibitory control have been proposed based on performance in other behavioral paradigms: MAM-E17 rats showed an increased number of responses, decreased efficiency and number of rewards earned on a differential reinforcement of low-rate (DRL-20) task, as well as a nonsignificant trend to make more premature responses in the 5-CSRTT (Featherstone et al. 2007). Moreover, the robust pattern of MAM-E17 deficits in reversal and extra-dimensional shift learning is suggestive of general impairments in the ability to flexibly inhibit responses to prepotent or behaviorally salient stimuli (Moore et al. 2006; Featherstone et al. 2007; Gastambide et al. 2012). Increased false alarm rates are also frequently reported in schizophrenic patients (Wohlberg and Kornetsky 1973; Birkett et al. 2007; Nuechterlein et al. 2015) and are found to be correlated with negative (Nuechterlein et al. 1986) or disorganized symptom domains of the disorder (Vollema and Postma 2002).

The observed deficits in attentional and inhibitory response control on the rCPT are predicted based on the known neurodevelopmental sequelae of the MAM-E17 model (Lodge and Grace 2007; Chen et al. 2014; Perez et al. 2014; Grace 2016; Penschuck et al. 2006; Lodge et al. 2009; Maćkowiak et al. 2014). However, despite its similarities to schizophrenia and robust performance impairments on the rCPT, it is noteworthy that previous studies have not readily observed specific deficits in attention and executive function within the MAM-E17 model. Impairments in prepulse inhibition, latent inhibition, set shifting, and learning or performance on spatial tasks are well documented in MAM-E17 animals (Flagstad et al. 2005; Moore et al. 2006; Featherstone et al. 2007; Gastambide et al. 2012; O’Reilly et al. 2016), but owing to factors such as hyperactivity and/or other behavioral processes, their precise links to attentional and/or executive control are unclear. Moreover, when the MAM-E17 model was evaluated on tasks explicitly designed to be analogous to human CPTs such as the 5-CSRTT or SAT/dSAT, no significant differences from sham controls were observed on any performance measures (Featherstone et al. 2007; Howe et al. 2015). The present rCPT was designed to incorporate key parameters that have been demonstrated to tax attentional and executive control processes in the human CPT paradigm. These parameters include the successive presentation of stimuli which demands withholding responses on nontarget trials, as well as the use of visual stimuli that require both detection and discrimination between target and nontargets matched for overall luminance (Parasuraman 1979; See et al. 1995). The 5-CSRTT, SAT/dSAT, and related paradigms do not require withholding responses on any trials and only require detection and/or localization of luminance increments. These task differences are likely to engage distinct neural mechanisms and might help explain the increased sensitivity of the rCPT to detect impairments in attentional and executive control in the MAM-E17 model. It should be noted that the present study examined stable rCPT performance in middle-aged, male rats to help make it comparable to previous studies examining attentional and executive performance in the MAM-E17 model. It would be worthwhile to extend investigations to include female subjects and to assess model differences at an earlier developmental period—from late juvenile to young adulthood—which represents a critical time for schizophrenia onset and which may also provide a better potential window for successful treatment intervention.

### Pharmacological effects of psychoactive compounds

#### Suppressant-like effects (sulpiride, atomoxetine, LSN2463359, RO4938581)

Acute administration of the D2/D3 receptor antagonist and neuroleptic, sulpiride (0–30 mg/kg); the selective noradrenergic reuptake inhibitor, atomoxetine (0–1 mg/kg); the mGlu_5_R PAM, LSN2463359 (0–5 mg/kg); and the GABA(A)_α5_R partial inverse agonist, RO4938581 (0–10 mg/kg), each exerted suppressant-like effects on rCPT performance by dose-dependently reducing indices of overall responding across all animals. These reductions were evidenced by an increased response criterion, *c*, and decreased ISI touches, HR, and/or FAR. Given that incorrect and reward collection latencies were unaltered by these compounds, the suppressant effects are unlikely to be simply attributed to potentially confounding factors such as motor depression or anhedonia. Moreover, with the exception of the highest dose of atomoxetine, the general absence of drug effects on sensitivity (*d*′) further suggests that the effects cannot be easily related to changes in sustained, focused attention or visual discrimination processes.

The suppressant-like effects of these compounds are consistent with numerous prior studies and may suggest that they are acting to reduce impulsive/motivated responding to salient cues predictive of reward. Numerous studies have described D2/D3 antagonist-induced decreases in operant responding for reward (Harrison et al. 1997; Heath et al. 2015; Froger-Colléaux and Castagné 2016). Anti-impulsive effects of atomoxetine have also been widely observed in both rodents and humans tested on a variety of cognitive paradigms (Blondeau and Dellu-Hagedorn 2007; Navarra et al. 2008; Robinson et al. 2008; Paterson et al. 2011; Fernando et al. 2011; Baarendse and Vanderschuren 2011; Robinson 2012; Chamberlain et al. 2006, 2007; Bari et al. 2009; Tomlinson et al. 2014).The mGlu_5_R PAM, ADX47273, has been observed to reduce the number of impulsive responses in normal healthy rats in the 5-CSRTT (Liu et al. 2008; Isherwood et al. 2015), and RO4938581, as well as several mGlu_5_R PAMs, has been demonstrated to reduce hyperactivity observed in models of NMDAr antagonist or psychostimulant administration (Schlumberger et al. 2010; Bartolomé-Nebreda et al. 2013; Conde-Ceide et al. 2016; Ballard et al. 2009; Redrobe et al. 2012).

None of these compounds exerted any beneficial effects on the rCPT performance sensitivity index, *d*′. For sulpiride, this is in line with the clinical literature indicating that current neuroleptic treatment mechanisms do not significantly improve fundamental attentional impairments in schizophrenia (Cornblatt et al. 1997; Nuechterlein et al. 2015). However, atomoxetine has enhanced performance accuracy on certain sustained attention assays, particularly when long and/or variable ITIs have been introduced or manipulated (Jentsch et al. 2008; Baarendse and Vanderschuren 2011; Robinson 2012; Tomlinson et al. 2014). Cognitive-enhancing effects of mGlu_5_R PAMs and GABA(A)_α5_R receptor-negative allosteric modulators (α5NAMs) have been reported on other tasks assessing behavioral flexibility, aversive learning, recognition memory, and working memory in normal, healthy mice and rats (Darrah et al. 2008; Uslaner et al. 2009; Stefani and Moghaddam 2010; Fowler et al. 2011; Gilmour et al. 2013; Dawson et al. 2006; Atack et al. 2006). Moreover, LSN2463359 (2.5 mg/kg) was observed to selectively restore impairments in reversal learning and extra-dimensional set shifting selectively in MAM-E17 rats in a bowl-digging paradigm (Gastambide et al. 2012). The putative anti-impulsive properties of these compounds may provide a parsimonious and testable explanation for the pattern of nootropic effects on certain other behavioral assays. Enhancement of task performance by compounds that help reduce impulsivity might be predicted within experimental paradigms in which rapid stimulus processing and responding are not required and where hyperactivity or impulsivity in model animals may confound and/or impair performance.

We observed detrimental effects of higher-dose (1.0 mg/kg) atomoxetine on *d*′. Such impairments may be predicted by inverted-U theories of catecholamine function (Aston-Jones and Cohen 2005; Robbins and Arnsten 2009) and have also been observed in primates in a working memory task (Gamo et al. 2010) and a decision-making task in rodents (Silveira et al. 2016). The novel features of the rCPT relative to other rodent attentional paradigms may render it more demanding and more sensitive to detrimental effects of higher doses of pro-catecholaminergic agents.

Sulpiride was the only drug to selectively alter rCPT performance of MAM-E17 animals by dose-dependently reducing their elevated false alarm rate. This result fits well with findings that sulpiride treatment can specifically remediate dysfunctional plasticity within hippocampal and frontostriatal circuitry of MAM-E17 rats (Belujon et al. 2013; Belujon et al. 2014). Moreover, antagonism of dopamine D2 and/or D3 receptors in the nucleus accumbens core has been demonstrated to selectively improve inhibitory control in other model animals (medial prefrontal cortex (mPFC) lesioned, amphetamine injected, or selected high-impulsive animals) that exhibit high levels of impulsivity on the 5-CSRTT (Pattij et al. 2006; Pezze et al. 2008; Besson et al. 2010). Systemic D2/D3 antagonism may reduce motivational salience for stimuli and responses governing rCPT performance (Treadway and Zald 2013) and preferentially remediate an inhibitory control deficit in MAM-E17 animals that may result from aberrant frontostriatal-based salience mechanisms, as hypothesized for schizophrenia (Kapur 2003).

#### Stimulant-like effects (modafinil, ABT-594)

The eugeroic agent, modafinil (8 mg/kg), was the only compound to cause significant improvements in sensitivity, *d*′, a common index of sustained, focused attentional performance in the CPT paradigm, but did so only in control rats. Modafinil has shown mixed effects for cognitive enhancements in healthy human subjects but generally improves performance on tests of sustained and focused attention at more difficult task parameters using low to moderate doses (Scoriels et al. 2013; Battleday and Brem 2015). While the majority of studies using young, healthy rodents in the 5-CSRTT has found either no effect or reductions in attentional accuracy and/or inhibitory control (Milstein et al. 2003; Waters et al. 2005; Liu et al. 2010), such results may be explained by the use of higher dose ranges (>30 mg/kg) and/or insufficient task difficulty. We also found that higher doses of modafinil in the rCPT increased FAR and ISI touches, decreased criterion (*c*), and a significant linear trend toward faster correct response latencies across all animals. Together with our observed impairments in *d*′ at the 64 mg/kg dose, these results suggest that the stimulant effects of modafinil at higher doses might be subject to a speed/accuracy tradeoff and be detrimental to task performance. Indeed, higher doses of modafinil (64–128 mg/kg) have been reported to induce locomotor hyperactivity in rats (Simon et al. 1995; McClellan and Spencer 1998). Similar to our rCPT results, low-dose modafinil (10 mg/kg) has been observed to improve performance on a stop signal reaction time task specifically in low-performing rats, while impairing behavior at higher 100 mg/kg doses (Eagle et al. 2007). Moreover, one study has reported an enhancement in attentional accuracy following repeated daily dosing of modafinil (8–64 mg/kg) in older rats having low baseline performance levels on a three-stimulus detection task with variable stimulus durations and inter-trial intervals (Morgan et al. 2007). Thus, both the lower dose (8 mg/kg) and the increased difficulty of the rCPT over the 5-CSRTT (e.g., variable stimulus durations and ITIs, plus discrimination versus simple detection) may have rendered it more sensitive for detecting the attention-enhancing effects of modafinil.

It is unclear why modafinil enhanced sham performance but was ineffective within the MAM-E17 model. One possibility is that disruptions of mPFC structure and function (Grace 2016), a brain region consistently implicated in successful CPT performance (Rosvold et al. 1956; Glosser and Goodglass 1990; Carter et al. 1998), might preclude the drugs’ beneficial effects. Modafinil has been demonstrated to increase dopamine levels (de Saint Hilaire et al. 2001) and activity (Gozzi et al. 2012) within the mPFC of the rat and to alter PFC activity during cognitive demand in humans (Rasetti et al. 2010; Minzenberg et al. 2010). Interestingly, although modafinil has been found to improve aspects of executive control (Turner et al. 2004), it has generally not been found to rectify sustained attentional deficits in schizophrenia (Sevy et al. 2005; Freudenreich et al. 2009; Kane et al. 2010). Further investigation into the mechanisms of modafinil on attention is warranted.

The selective α_4_β_2_nAChR agonist ABT-594 exerted stimulant-like effects on HR, FAR, *c*, ISI touches, and correct response latency across both groups of animals on the rCPT. Stimulant effects of α4β2 agonists have been observed in a variety of tasks including locomotor activity (Bannon et al. 1998) and the 5-CSRTT (Grottick and Higgins 2000; Hahn et al. 2003; Mohler et al. 2010). We did not see any ABT-594-related improvements in *d*′, which suggests that the drug is not augmenting the sustained capacity to correctly discriminate target from nontarget stimuli. However, our observed increases in HR and rewards earned and decreases in correct response latency are potential indices of cognitive enhancement and consistent with effects observed in other attentional paradigms. Improvements in response accuracy, total correct, and speeded correct reaction times have also been observed under specific conditions within the 5-CSRTT and SAT/dSAT paradigms following α_4_β_2_nAChR agonist treatment (McGaughy et al. 1999; Mohler et al. 2010; Howe et al. 2010). This pattern of results might be due to α4β2 agonists acting to lower the threshold for *go* responses to visually detected stimuli (presence or absence of light). This hypothesis would explain the selective improvements in performance accuracy on detection tasks only when baseline performance is low. Moreover, as *d*′ on the rCPT requires sustained attention for both visual discrimination and visual detection processes, it may be that the former is not improved by nicotine or α_4_β_2_nAChR agonists. This interpretation might also fit with human studies showing faster responding but inefficacy of nicotine against the sustained attention deficits induced by ketamine in humans (D’Souza et al. 2012) and failure of the α_4_β_2_nAChR agonist AZD3480 to improve sustained attention in schizophrenic patients when visual discrimination processes are required (Velligan et al. 2012).

#### Compounds with limited effects on rCPT performance (donepezil, EVP-6124)

We observed no significant effects of the cholinesterase inhibitor, donepezil (0–1.0 mg/kg), on any measure of rCPT performance in MAM-E17 or sham controls. This result is consistent with recent reviews and large meta-analyses, suggesting that the impact of donepezil on sustained attentional performance in schizophrenia is equivocal (Singh et al. 2012; Thakurathi et al. 2013; Choi et al. 2013). There are also limited effects of donepezil on attention in healthy individuals (Beglinger et al. 2004). Our data did, however, show a near-significant effect of donepezil dose on *d*′ across all animals (*p* = 0.064)—strongest at the lowest 0.1 mg/kg dose—but also show mean improvements at 0.3 and 1.0 mg/kg relative to vehicle. Donepezil is known to enhance behavioral contrast sensitivity, particularly within sensitive spatial frequency ranges (Soma et al. 2013). Moreover, we have recently observed that donepezil can enhance *d*′ of DBA mice in the rCPT paradigm, specifically on more difficult, shorter-stimulus durations (Kim et al. 2015). The enhancing effects of donepezil on sustained and focused visual attention may thus depend critically on the visual capacity of the subject and the visual properties of the stimuli used. It would be worthwhile to examine the effects of donepezil or other pro-cholinergic compounds both in the MAM-E17 model and in schizophrenia patients using a CPT-like paradigm in which task parameters such as contrast, spatial frequency, and stimulus duration are expressly manipulated.

The partial α7nAChR agonist, EVP-6124 (0–3.0 mg/kg), did not affect rCPT performance in sham controls or MAM-E17 animals. There has been much clinical interest around the therapeutic potential of α7nAChR stimulation (Garay et al. 2016), and one recent study showed that EVP-6124 (1.0 mg/kg) improved attentional control selectively in a low-performing subgroup of rats using the 5C-CPT (Hayward et al. 2017). However, acute α7nAChR administration generally does not affect rodent attentional performance as measured by the 5-CSRTT (Grottick et al. 2003; Hahn et al. 2003; Hoyle et al. 2006; Nilsson et al. 2016). Moreover, α7nAChR agonists have repeatedly demonstrated nonsignificant effects on sustained attention in individuals with schizophrenia (Olincy et al. 2006; Freedman et al. 2008; Lieberman et al. 2013; Preskorn et al. 2014; Umbricht et al. 2014). EVP-6124 has recently failed a phase III trial with the endpoint of improved cognitive function in schizophrenia due to a lack of efficacy (Fidler 2016). Thus, our lack of enhancing effects of EVP-6124 on attention and executive function appears to be in general agreement with the bulk of preclinical and clinical findings.

## Summary and conclusions

MAM-E17 rats show a robust and reproducible pattern of deficits on a novel touchscreen continuous performance test that closely mirrors impairments observed in schizophrenia and other disorders (Table 1). The specific deficits in sustained attention/executive control found using the rCPT paradigm are predicted based on underlying neurobiological disruptions in the MAM-E17 model yet are the first to be unambiguously detected within a rodent behavioral paradigm.

The eight acutely administered compounds tested in this study each exhibited distinct behavioral profiles on rCPT performance variables as summarized in Table 2. Sulpiride, atomoxetine, LSN2463359, and RO493858 each showed suppressant effects on rCPT performance, including increases in response criterion (*c*) and decreases in ISI touches and HR and/or FAR. Conversely, modafinil and ABT-594 exerted stimulant-like effects with decreases in *c* and increases in ISI touches, HR, and/or FAR. Donepezil showed near-significant enhancements in *d*′, and EVP-6124 exerted no effects on the rCPT. In terms of model-specific drug effects, sulpiride dose-dependently reduced the elevated FAR in MAM-E17 rats whereas low-dose modafinil (8 mg/kg) improved *d*′ only in sham controls. These drug profiles are largely consistent with the human and rodent literatures using similar attentional paradigms and highlight their observed general lack of effectiveness for selectively improving *d*′ in schizophrenia. The rCPT may be an important translational test for the human CPT paradigm, which is one of the most widely used tools for the assessment of sustained and focused attention and executive function in clinical neuropsychometrics.

## Electronic supplementary material

ESM 1 (DOCX 116 kb)
