# Supplementary material for: MAM-E17 rat model impairments on a novel continuous performance task: effects of potential cognitive enhancing drugs
Source: Psychopharmacology (Berl). 2017 Jul 26;234(19):2837–57. doi: 10.1007/s00213-017-4679-5 (PMC5591806; doi:10.1007/s00213-017-4679-5)
Supplement: Supplementary file 1 — (DOCX 116 kb) [file 213_2017_4679_MOESM1_ESM.docx]

**SUPPLEMENTARY MATERIAL**

MAM-E17 rat model impairments on a novel continuous performance task: Effects of potential cognitive enhancing drugs

Adam C Mar^1^*^,2,^*^3,4*^, Simon RO Nilsson^1,2,3,4^, Begoña Gamallo-Lana^1,2,3,4^, Ming Lei^3,4,5^, Theda Dourado^3,4^, Johan Alsiö^3,4,6^, Lisa M Saksida^3,4,7,8^, Timothy J Bussey^3,4,7,8^ & Trevor W Robbins^3,4^

^1^Neuroscience Institute, New York University Medical Center, New York, NY, USA.

^2^Department of Neuroscience and Physiology, New York University Medical Center, New York, NY, USA.

^3^Department of Psychology, University of Cambridge, Cambridge, UK.

^4^MRC and Wellcome Trust Behavioural and Clinical Neuroscience Institute, University of Cambridge, Cambridge, UK.

^5^Department of Health Industry Management, Beijing International Studies University, 1 Dingfuzhuang Nanli, Beijing, China.

^6^Department of Neuroscience, Unit of Functional Neurobiology, University of Uppsala, Uppsala, Sweden.

^7^Molecular Medicine Research Group, Robarts Research Institute & Department of Physiology and Pharmacology, Schulich School of Medicine & Dentistry, Western University, London, ON, Canada. ^8^The Brain and Mind Institute, Western University, London, ON, Canada.

*Corresponding author:* Adam C Mar, Department of Neuroscience and Physiology, Neuroscience Institute, New York University Medical Center, New York, NY, 10016, USA. Email: adam.mar@nyumc.org. Tel: +1-212-263-9295. Fax: +1-212-263-9120

Keywords: Attention, MAM-E17, Executive function, Touch screen, Behavioral Pharmacology, Schizophrenia, ADHD, Animal model

SUPPLEMENTARY RESULTS

**Effect of MAM-E17 on rCPT performance**

The consistency of the MAM-E17 model differences was also preserved across many of the Latin square dosing regimens for the administered compounds. For the performance sensitivity index, d’, there were main effects of Group – with MAM-E17 rats having decreased d’ relative to sham controls – during RO493858 (F[1,42] = 9.105, p = 0.004), donepezil (F[1,27] = 6.964, p = 0.014), ABT-594 (F[1,29] = 15.798, p < 0.001), atomoxetine (F[1,29] = 10.586, p = 0.003), and sulpiride (F[1,28] = 11.127, p = 0.002) administration. Non-significant decreases in d’ of MAM-E17 animals were also observed during the EVP-6124 (p = 0.168), LSN2463359 (p = 0.055), modafinil (p = 0.153) experiments (Figs. 2-4). MAM-E17 animals showed significantly increased FAR compared to sham controls during LSN2463359 (F[1,15] = 5.837, p = 0.029), RO493858 (F[1,43] = 16.528, p < 0.001), donepezil (F[1,27] = 6.817, p = 0.015), ABT-594 (F[1,29] = 11.140, p = 0.002), and sulpiride (F[1,28] = 8.088, p = 0.008) dosing experiments, as well as non-significant trends toward increases in FAR when dosed with EVP-6124 (p = 0.090), modafinil (p = 0.083) and atomoxetine (p = 0.052) (Figs. 2-4). Moreover, MAM-E17 animals also showed significant increases in ISI touches relative to sham controls during RO493858 (F[1,43] = 9.485, p = 0.004), ABT-594 (F[1,29] = 5.104, p = 0.032) and sulpiride (F[1,28] = 4.223, p = 0.049) experiments, with non-significant increases observed for EVP-6124 (p = 0.121), LSN2463359 (p = 0.056), modafinil (p = 157), donepezil (p = 0.094) and atomoxetine (p = 0.114) (Figs. 2-4). There were no main effects of Group on measures of HR, response criterion c, or on any of the latency indices across the eight drug dosing experiments.

**Effect of acute drug treatments on rCPT performance**

The nonsignifcant statistical results for each rCPT measure, by drug Latin Square, are presented below. All of the means and standard errors for each primary rCPT measure at each drug dose are presented in Supplementary Table 1.

*Sulpiride.* Sulpiride treatment did not significantly alter performance sensitivity, d’, in the rCPT (Dose: F[3.84] = 1.320, p = 0.273; Dose x Group: F[3,84] = 0.797, p=0.499). There were no significant effects of sulpiride on incorrect response latency (Dose: F[3.84] = 1.136, p = 0.338; Dose x Group: F[3,84] = 1.416, p=0.246 – H-F corrected) or reward collection latency (Dose: F[3.84] = 0.747, p = 0.433; Dose x Group: F[3,84] = 1.426, p=0.248 – H-F corrected).

*Atomoxetine.* There was no effect of atomoxetine on reward collection latency (Dose: F[3.87] = 0.362, p = 0.615; Dose x Group: F[3,87] = 0.429, p=0.576 – H-F corrected).

*LSN2463359*. LSN2463359 treatment did not significantly alter performance sensitivity, d’, in the rCPT (Dose: F[3,45] = 2.149, p = 0.133; Dose x Group: F[3,45] = 0.019, p = 0.983 – H-F corrected). There was also a non-significant trend for higher LSN2463359 doses to reduce FAR (Dose: F[3,45] = 2.395, p = 0.081; Dose x Group: F[3,45] = 0.159, p = 0.923). There were no significant effects of LSN2463359 on correct response latency (Dose: F[3,45] = 1.865, p = 0.149; Dose x Group: F[3,45] = 0.250, p = 0.861) or incorrect response latency (Dose: F[3,45] = 2.312, p = 0.089; Dose x Group: F[3,45] = 2.170, p = 0.105). There was a significant Dose x Group interaction for reward collection latency (Dose: F[3,42] = 2.110, p = 0.144; Dose x Group: F[3,42] = 3.609, p = 0.045 – H-F corrected), but post hoc tests revealed no significant pairwise comparisons.

*RO4938581.* RO4938581 treatment did not significantly alter performance sensitivity, d’, in the rCPT (Dose: F[3,126] = 0.812, p = 0.489; Dose x Group: F[3,126] = 0.876, p = 0.455). There were no significant effects of RO4938581 on incorrect response latency (Dose: F[3,126] = 0.917, p = 0.435; Dose x Group: F[3,126] = 2.652, p = 0.052) or reward collection latency (Dose: F[3,126] = 0.426, p = 0.652; Dose x Group: F[3,126] = 1.248, p = 0.292 – H-F corrected).

*Modafinil.* There was no significant effect of modafinil on HR (Dose: F[3,51] = 0.390, p = 0.735; Dose x Group: F[3,51] = 1.241, p = 0.304 – H-F corrected). There was no significant effect of modafinil on correct response latency (Dose: F[3,51] = 1.938, p = 0.135; Dose x Group: F[3,51] = 0.136, p = 0.938), incorrect response latency (Dose: F[3,51] = 1.630, p = 0.194; Dose x Group: F[3,51] = 1.252, p = 0.301) or reward collection latency (Dose: F[3,51] = 0.954, p = 0.408; Dose x Group: F[3,51] = 1.918, p = 0.152 – H-F corrected). However, within-subjects trend analysis did reveal a significant linear relationship where higher modafinil doses were associated with faster correct response latencies (F[1,17] = 8.367, p < 0.010).

*ABT-594.* In the Latin square, ABT-594 treatment did not significantly alter performance sensitivity, d’, in the rCPT (Dose: F[3.87] = 1.057, p = 0.372; Dose x Group: F[3,87] = 0.500, p=0.683 – H-F corrected). There was a significant Dose x Group interaction on incorrect response latency (Dose: F[3.87] = 0.309, p = 0.819; Dose x Group: F[3,87] = 3.356, p=0.022), but post hoc tests revealed no significant pairwise comparisons. There was no significant effect of ABT-594 on reward collection latency (Dose: F[3.87] = 0.719, p = 0.429; Dose x Group: F[3,87] = 1.742, p=0.196 – H-F corrected).

*Donepezil.* There was a non-significant trend for a main effect of donepezil to enhance d’ performance across both MAM-E17 and sham-control rats (Dose: F[3,81] = 2.584, p = 0.064; Dose x Group: F[3,81] = 1.256, p < 0.295 – H-F corrected). This trend toward performance enhancement was most pronounced at the lowest 0.1 mg/kg donepezil dose relative to vehicle treatment. There were no significant effects of donepezil on any other primary performance measure in the rCPT. Specifically, there were no significant effects of donepezil on HR (Dose: F[3,81] = 1.120, p = 0.346; Dose × Group: F[3,81] = 0.080, p = 0.970), FAR (Dose: F[3,81] = 0.748, p = 0.527; Dose × Group: F[3,81] = 1.771, p = 0.159), c (Dose: F[3,81] = 0.354, p = 0.786; Dose × Group: F[3,81] = 0.201, p = 0.896) or ISI touches (Dose: F[3,81] = 0.832, p = 0.480; Dose × Group: F[3,81] = 1.882, p = 0.139). There were also no significant effects of donepezil correct response latency (Dose: F[3.81] = 0.481, p = 0.674; Dose x Group: F[3,81] = 0.731, p=0.522 – H-F corrected), incorrect response latency (Dose: F[3.81] = 0.637, p = 0.594; Dose x Group: F[3,81] = 0.810, p=0.492) or reward collection latency (Dose: F[3,81] = 2.111, p = 0.133; Dose x Group: F[3,81] = 2.116, p = 0.133 – H-F corrected).

*EVP6124.* EVP6124 did not alter any of the primary performance measures in the rCPT. There were no significant effects of EVP6124 on d’ (Dose: F[3,48] = 0.587, p = 0.578; Dose × Group: F[3,48] = 0.826, p = 0.457 – H-F corrected), HR (Dose: F[3,51] = 0.049, p = 0.986; Dose × Group: F[3,51] = 0.499, p = 0.685), FAR (Dose: F[3,51] = 0.500, p = 0.626; Dose × Group: F[3,51] = .935, p = 0.409 – H-F corrected), c (Dose: F[3,48] = 0.306, p = 0.821; Dose × Group: F[3,48] = 0.243, p = 0.866) or ISI touches (Dose: F[3,51] = 0.985, p = 0.368; Dose × Group: F[3,51] = 0.306, p = 0.687 – H-F corrected). There was a near-threshold significant main effect of EVP6124 dose on correct response latency (Dose: F[3,48] = 2.776, p = 0.050; Dose x Group: F[3,48] = 1.346, p=0.270), where the largest effect was slower correct response times at the 1 mg/kg dose . There were no significant effects of EVP6124 on incorrect response latency (Dose: F[3.48] = 2.117, p = 0.132; Dose x Group: F[3,48] = 1.905, p=0.161 – H-F corrected) or reward collection latency (Dose: F[3,48] = 1.500, p = 0.240; Dose x Group: F[3,48] = 1.421, p = 0.253 – H-F corrected)

|  | *p <* |  |
| --- | --- | --- |
|  | 0.05 | Sig. increase from vehicle |
|  | 0.01 |  |
|  | 0.001 |  |
|  |  |  |
|  | 0.05 | Sig. decrease from vehicle |
|  | 0.01 |  |
|  | 0.001 |  |
|  |  |  |
|  | 0.05 | Sig. group x |
|  |  | dose |
|  |  | interaction |

*Note.* Significant effects of group denoted by bold and bordered by broken line. Effects of drug dose and group x dose interactions denoted by colour with statistical significance highlighted by colour code, please see legend.

**Table S1.** Performance of MAM-E17 and sham animals in the rCPT during acute systemic pharmacological challenges.
